# Impact of adverse events on patient outcomes in a Japanese intensive care unit: a retrospective observational study

**DOI:** 10.1002/nop2.1040

**Published:** 2021-08-17

**Authors:** Gen Aikawa, Akira Ouchi, Hideaki Sakuramoto, Chiemi Ono, Chie Hatozaki, Mayu Okamoto, Tetsuya Hoshino, Nobutake Shimojo, Yoshiaki Inoue

**Affiliations:** ^1^ Department of Emergency and Critical Care Medicine Faculty of Medicine University of Tsukuba Tsukuba Japan; ^2^ Intensive Care Unit University of Tsukuba Hospital Tsukuba Japan; ^3^ Department of Adult Health Nursing College of Nursing Ibaraki Christian University Hitachi Japan

**Keywords:** adverse event, global trigger tool, intensive care, patient safety

## Abstract

**Aim:**

We investigated adverse events (AEs) in a Japanese intensive care unit (ICU) and evaluated the impact of cause‐specific AEs on mortality and length of stay.

**Design:**

A retrospective observational study in the ICU of an academic hospital.

**Methods:**

We reviewed medical records with the Global Trigger Tool.

**Results:**

Of the 246 patients, 126 (51%) experienced one or more AEs with an incidence of 201 per 1000 patient‐days and 115 per 100 admissions. A total of 294 AEs were detected with 119 (42%) adverse drug events, 67 (24%) procedural complications, 63 (22%) surgical complications, 26 (9%) nosocomial infections, 5 (2%) therapeutic errors and 4 (1%) diagnostic errors. Adverse event (AE) presence was associated with length of ICU stay (β = 2.85, 95% confidence interval [CI]: 1.09–4.61). Adverse drug events, procedural complications and nosocomial infections were strongly associated with length of ICU stay (β = 2.38, 95% CI: 0.77–3.98; β = 3.75, 95% CI: 2.03–5.48; β = 6.52, 95% CI: 4.07–8.97 respectively).

## 1 INTRODUCTION

Adverse events (AEs) are defined as “unintended physical injury resulting from or contributed to by medical care that requires additional monitoring, treatment or hospitalization or that results in death (Griffin & Resar, 2009).” Diverse studies from various countries reported that AEs developed in 12% of hospitalized patients (Panagioti et al., 2019) while the severe and unstable patients often seen in the intensive care unit (ICU) experienced more AEs than those in other general wards (Andrews et al., 1997). Up to 20%–25% of ICU patients experience an adverse event (AE), with 45.3–80.5 events per 1000 patient‐days, and, within these events, 13% were lethal or life‐threatening (Rothschild et al., 2005; Sauro et al., 2020). Numerous international studies show that AEs increase ICU stay length by 8.9 days and the length of a hospital stay by 6.8 days (Ahmed et al., 2015).

The incidence of AEs varies according to national background and medical culture. For example, anaesthesia‐related mortality is higher in developing countries than in developed countries (Bainbridge et al., 2012). Also, patient safety culture scores have been found to be negatively correlated with AEs incidence (Han et al., 2020; Mardon et al., 2010; Najjar et al., 2015), and Japan was reported to have a lower score for patient safety culture than the United States (Fujita et al., 2013). In Japan, medical policy promotes an error‐based incident reporting system, but, on the other hand, few studies have focussed on AEs that are important for patients. Additionally, most of these studies are limited to drug‐related AEs and do not report AEs within targeted ICU populations (Anzai et al., 2019; Chisaki et al., 2017; Fujiwara et al., 2016; Hatahira et al., 2018; Matsumura et al., 2018; Suga et al., 2019; Tsuchiya et al., 2020).

Furthermore, AEs may be subclassified by cause, such as adverse drug events, surgical complications, procedural complications, nosocomial infections and error (Forster et al., 2008). However, many studies have integrated and analysed AEs without comparing cause which obscures cause‐specific AE impact on patient outcomes (Ahmed et al., 2015).

Therefore, the purpose of the present study was to investigate AEs in a Japanese general ICU and evaluate the impact of cause‐specific AEs on patient outcomes.

## 2 METHODS

### 2.1 Study design

We retrospectively reviewed electronic medical records with the Global Trigger Tool (GTT) developed by the Institute for Healthcare Improvement to detect AEs. Trigger tool methodology is a retrospective review of a random sample of medical records with triggers to identify possible AEs. Examples of triggers include acute dialysis, pneumonia onset or intubation. GTT consists of 53 triggers defined in six different modules (cares, medication, surgical, intensive care, perinatal and emergency department). When a trigger was found, a careful analysis was conducted to confirm whether an AE was related to the trigger event. The following definitions of AEs were used: unintended physical injury resulting from or contributed to by medical care that requires additional monitoring, treatment or hospitalization or that results in death. Psychological harm (by definition) is excluded as an AE in the GTT, which focusses on AEs related to the active delivery of care (commission) and excludes issues related to substandard care (omission) (Griffin & Resar, 2009).

### 2.2 Setting

The study was conducted in the ICU of an 800‐bed academic hospital in Japan. The unit itself is a general ICU with twelve beds and approximately 700–800 admitted patients per year. The system is an open ICU with an ICU nursing staff‐to‐patient ratio of 1:2.

### 2.3 Record selection

For each month, 20 medical admission records were randomly selected between April 2016 and March 2017 in ICU using “RANDBETWEEN” as a randomization function (Griffin & Resar, 2009). Exclusion criteria were (a) under the age of 18 years, (b) a length of stay less than 24 hr or (c) readmission. Short stays were excluded because of a lack of information by which to determine AEs.

### 2.4 Data collection

Patient data were retrospectively collected from electronic medical records. Demographic data collected include age, gender, the disease for ICU admission, mechanical ventilation status, admission category (medical, elective surgery, emergency surgery), location prior to ICU and Acute Physiology and Chronic Health Evaluation II (APACHE II) score (scale range, 0–71) as a marker of illness severity. The APACHE II score is calculated by the most abnormal values obtained during the first 24 hr of an ICU stay (Knaus et al., 1985). Outcome data collected include 28‐day mortality, hospital mortality, length of ICU stay and length of hospital stay.

### 2.5 Review process

The review team consisted of two registered intensive care nurses (primary reviewers) and one intensivist (secondary reviewer). All reviewers had more than 5 years of ICU experience and general knowledge about the ICU. The review process was performed in two‐stages following GTT guidelines (Griffin & Resar, 2009). In stage 1, primary reviewers used GTT to independently conduct reviews of individual electronic medical records within 20 min or less. All reviews were conducted manually by looking through text fields that included the physician's diagnosis, treatment records, surgical records and nursing care records from ICU admission until 2 days after ICU discharge to clarify AEs occurring in the ICU. The primary reviewer screened for one or more of the 53 triggers then marked any such triggers on the GTT worksheet and described suspected AEs with a one‐ to two‐paragraph summary. Primary reviewers then compared findings and came to a consensus.

In stage 2, the secondary reviewer did not review patient medical records directly but only performed a review of the primary reviewers' summaries. Any suspected AE for which the secondary reviewer disagreed was discussed, and a final consensus was reached on the presence, severity and preventability of suspected AEs.

Since review training was not mentioned in the Japanese GTT, the GTT manual was distributed to the reviewers and, after one training session, we started the review.

### 2.6 Preventability and severity of adverse events

Preventability of AEs was assessed as “an error in management due to failure to follow accepted practice at an individual or system level (Rodziewicz et al., 2021).” The degree of preventability was scored on a modified three‐grade scale: grade 1 was “virtually no evidence for preventability,” grade 2 was “preventability not likely, less than 50%,” and grade 3 was “preventability more likely than not, more than 50%.” AEs were judged to be preventable if scored as grade 3 (Schwendimann et al., 2018).

Adverse event severity was categorized according to the Medication Error Index adopted by the National Coordinating Council for Medication Error Reporting and Prevention with categories between E and I (National Coordinating Council for Medication Error Reporting & Prevention, 2001). Category E is temporary harm requiring intervention, category F is temporary harm requiring initial or prolonged hospitalization, category G is permanent harm, category H is life‐threatening harm, and category I is harm causing or contributing to death.

### 2.7 Statistical analysis

Quantitative variables are reported as median (interquartile range, IQR) and qualitative variables as number (%). The incidence of the AE rate was calculated as the number of AEs per 1000 patient‐days and per 100 admissions.

Characteristics and outcomes were compared using the Mann–Whitney *U* test for quantitative variables and Pearson's chi‐squared test or Fisher's exact test for qualitative variables. Associations between the presence of the AE and patient outcome (28‐day mortality, hospital mortality, length of ICU stay and length of hospital stay) were examined using linear regression analysis or logistic regression analysis, and results are reported as coefficients with 95% confidence intervals. These multivariate analysis models included the following covariates most likely to affect patient outcomes: presence of the AE, APACHE II score and mechanical ventilation required. The *p* values <.05 indicated statistical significance. All analyses were conducted with IBM SPSS Statistics version 25 (IBM Corp.).

### 2.8 Ethics approval

The institutional review board of our hospital approved this study (H30‐126). The need for informed consent from each patient was waived due to the retrospective design and use of anonymized patient data.

## 3 RESULTS

### 3.1 Characteristics and patient outcomes

**FIGURE 1:**
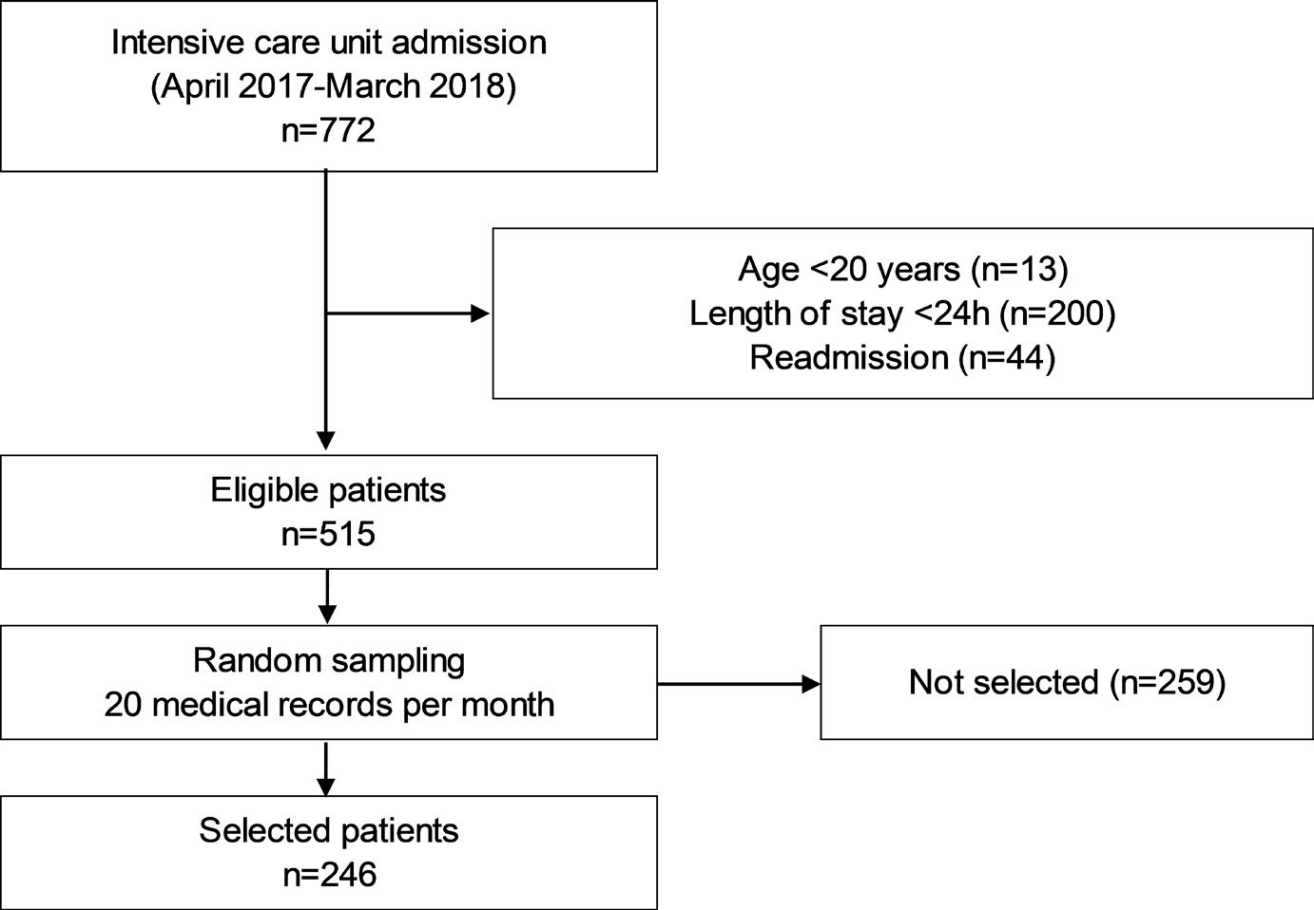
Flow chart of the study population

Out of 772 patients admitted to the ICU within the study period, 257 patients were excluded while the remaining 515 eligible patients were randomly sampled at 20 per month. Finally, 246 patients were selected (Figure 1).

Patient characteristics and outcomes are given in Table 1. The median age was 68 (55–75) years, 157 (64%) patients were male, 128 (52%) patients required mechanical ventilation, and the median APACHE II score was 13 (9–20). Patients were divided into two groups based on if they had one or more AEs. Significant differences were found in mechanical ventilation status, APACHE II score, neuromuscular, trauma, 28‐day mortality, hospital mortality, length of ICU stay and length of hospital stay.

**TABLE 1:** Characteristics and outcomes of the patients

Variable	All patients (*N* = 246)		With AE (*N* = 126)		Without AE (*N* = 120)		*p*
Age, median (range)	68	(55–75)	69	(56–75)	68	(55–75)	.84
Male, *N* (%)	157	(64)	79	(62)	78	(66)	.60
Mechanical ventilation status, *N* (%)	128	(52)	99	(78)	29	(24)	<.01
APACHE Ⅱ score, median (range)	13	(9–20)	16	(11–23)	11	(8–15)	<.01
Urgently admitted patients, *N* (%)	124	(50)	61	(48)	63	(50)	.53
Admission category, *N* (%)							
Medical	107	(44)	49	(39)	58	(48)	.14
Elective surgery	114	(46)	61	(48)	53	(45)	.50
Emergency surgery	25	(10)	16	(13)	9	(8)	.18
Location prior to ICU admission, *N* (%)							
Surgical room	125	(51)	66	(52)	58	(49)	.53
Emergency department	91	(37)	45	(36)	48	(40)	.49
General ward	22	(9)	12	(10)	9	(8)	.57
Outside hospital	8	(3)	3	(2)	5	(4)	.49^a^
Disease for ICU admission, *N* (%)							
Cardiovascular	119	(48)	66	(52)	53	(45)	.20
Gastroenterology	28	(11)	14	(11)	14	(12)	.89
Neuromuscular	25	(10)	7	(6)	18	(15)	.01
Respiratory	15	(6)	9	(7)	6	(5)	.48
Cardiopulmonary arrest	13	(5)	9	(7)	4	(3)	.18
Trauma	9	(4)	8	(6)	1	(1)	.04^a^
Renal urology	8	(3)	2	(2)	6	(5)	.16^a^
Otorhinolaryngology	8	(3)	2	(2)	6	(5)	.16^a^
Shock	7	(3)	5	(4)	2	(2)	.45^a^
Sepsis	5	(2)	2	(2)	3	(3)	.68^a^
Drug overdose and poisoning	3	(1)	1	(1)	2	(2)	.61^a^
Other	6	(2)	1	(1)	5	(4)	.11^a^
28‐day mortality, *N* (%)	17	(7)	14	(11)	3	(3)	<.01
Hospital mortality, *N* (%)	28	(11)	22	(17)	6	(5)	<.01
Length of ICU stay, median (range)	3	(2–6)	5	(3–10)	3	(2–3)	<.01
Length of hospital stay, median (range)	22	(14–38)	24	(17–43)	18	(12–31)	<.01

Abbreviations: AE, adverse event; APACHE, Acute Physiology and Chronic Health Evaluation; ICU, intensive care unit.

^a^ The Fisher exact test was performed. All other binary variables were subjected to the chi‐square test.

### 3.2 Incidence and category of adverse events

A total of 246 patients' electronical medical records were reviewed. We identified 284 AEs and judged that 56 (20%) AEs were preventable. The changes over time per month in the incidence of AEs and disease severity are shown in Appendix 1. Of the total, 126 (51%) patients experienced one or more AEs and incidence was 201 events per 1000 patient‐days and 115 events per 100 admissions. According to severity, 185 (65%) events were category E, 87 (31%) events were category F, 2 (1%) events were category G, 6 (2%) events were category H, and 4 (1%) events were category I (Appendix 2). Some examples of AEs with high severity are shown in Appendix 3.

Among the 284 AEs, 119 (42%) were drug‐related (adverse drug event), 67 (24%) were from a procedure (procedural complication), 63 (22%) were from surgery (surgical complication), 26 (9%) were from infection (nosocomial infection), 5 (2%) were from therapeutic errors, and 4 (1%) were from diagnostic errors. The most frequent AEs were delirium in adverse drug events, skin tears in procedural complications and postoperative haemorrhaging in surgical complications (Appendix 4).

### 3.3 Association between adverse events and patient outcomes

Presence of an AE was strongly associated with length of ICU stay after adjusting for APACHE II score and mechanical ventilation status (β = 2.85, 95% CI: 1.09–4.61) but was not associated with 28‐day mortality, hospital mortality or length of hospital stay (Tables 2 and 3).

**TABLE 2:** Multivariate analysis of factors associated with mortality

Variable	OR (95% CI)	*p*
Model 1:28‐day mortality		
Presence of AE	0.53 (0.11–2.42)	.41
APACHE II score	1.20 (1.10–1.31)	<.01
Mechanical ventilation status	0.44 (0.04–4.78)	.50
Neuromuscular	0.44 (0.04–4.80)	.50
Trauma	0.65 (0.06–7.00)	.72
Model 2: Hospital mortality		
Presence of AE	0.69 (0.23–2.10)	.51
APACHE II score	1.11 (1.04–1.17)	<.01
Mechanical ventilation status	0.69 (0.23–0.97)	<.05
Neuromuscular	0.39 (0.07–2.39)	.31
Trauma	0.56 (0.09–3.30)	.52

Abbreviations: AE, adverse event; APACHE, Acute Physiology and Chronic Health Evaluation; CI, confidence interval; OR, odds ratio.

The mortality was a binary variable, and logistic regression analysis was performed and values were expressed as ORs.

**TABLE 3:** Multivariate analysis of factors associated with length of stay

Variable	β (95% CI)	*p*
Model 1: Length of ICU stay		
Presence of AE	2.85 (1.09–4.61)	<.01
APACHE II score	0.14 (0.03–0.25)	.02
Mechanical ventilation status	1.67 (−0.28–3.63)	.09
Neuromuscular	−0.31 (−3.02–2.42)	.83
Trauma	4.99 (1.05–8.92)	.01
Model 2: Length of hospital stay		
Presence of AE	−1.97 (−11.85–7.92)	.51
APACHE II score	0.74 (0.10–1.37)	.02
Mechanical ventilation status	7.77 (−3.21–18.75)	.17
Neuromuscular	2.33 (−12.96–17.62)	.76
Trauma	53.88 (31.78–75.99)	<.01

Abbreviations: AE, adverse event; APACHE, Acute Physiology and Chronic Health Evaluation; CI, confidence interval; ICU, intensive care unit.

Length of stay was a continuous variable, and linear regression analysis was performed and values were expressed as regression coefficient β.

The impact of cause‐specific AE classification on length of ICU stay is shown in Table 4. Adverse drug events, procedural complications and nosocomial infections were associated with length of ICU stay (β = 2.38, 95% CI: 0.77–3.98; β = 3.75, 95% CI: 2.03–5.48; β = 6.52, 95% CI: 4.07–8.97 respectively).

**TABLE 4:** Impact of cause‐specific adverse event on length of intensive care unit stay

Variable	β (95% CI)	*p*
Adverse drug event	2.38 (0.77–3.98)	<.01
Procedural complication	3.75 (2.03–5.48)	<.01
Surgical complication	1.29 (−0.68–3.26)	.20
Nosocomial infection	6.52 (4.07–8.97)	<.01
Therapeutic error	3.03 (−4.35–5.20)	.86
Diagnostic error	3.03 (−3.08–9.14)	.33
APACHE II score	0.06 (−0.04–0.17)	.24
Mechanical ventilation status	0.69 (−1.15–2.54)	.46
Neuromuscular	−0.02 (−2.25–2.20)	.98
Trauma	2.84 (−0.79–6.47)	.13

Abbreviations: APACHE, Acute Physiology and Chronic Health Evaluation; CI, confidence interval.

## 4 DISCUSSION

We found that 51% of ICU patients suffered from an adverse event, 20% of which were preventable. Most AEs were classified as “temporary harm” and became non‐preventable as the severity increased. Compared to a prospective study with direct ICU observation that reported an AE incidence of 20% at a rate of 80.5 events per 1000 patient‐days (Rothschild et al., 2005), our study had a higher frequency. As Japanese medical care standards are roughly equivalent to those in North America (GBD 2015 Healthcare Access & Quality Collaborators, 2017), any discrepancies may be attributed to differences in AE definitions between studies (those using trigger tools and those that do not) as well as inclusion of temporary and low‐impact events such as category E. Our results are in line with the largest previous study that used a trigger tool (ICU AE incidence of 55%, with 164 [range 60–200] events per 1000 patient‐days) (Resar et al., 2006). In terms of disease classification, AEs were found to be less frequent in neuromuscular (7/25:28%) and more frequent in trauma (8/9:89%) categories, in line with previous studies reporting AEs incidence in 24% of stroke and 29% of trauma patients (Daud‐Gallotti et al., 2005; Forster et al., 2008). The incidence of AEs in neurology patients was similar but more common in trauma patients. The reason for this is unclear, but we cannot rule out the possibility that, in addition to the aforementioned reasons, it was a coincidence due to the small case number.

In our study, AEs were not associated with mortality but were associated with length of ICU stay. Although AE occurrence has been reported to increase mortality (Roque et al., 2016; Sauro et al., 2020), meta‐analysis consistently shows that the AE is not statistically relevant (Ahmed et al., 2015). The relationship between the AE and length of ICU stay may display reverse causality, where a longer length of ICU stay increases the chance of AEs, but this is probably because AEs trigger the need for additional treatment. As low‐impact AEs, such as categories E and F, accounted for 96% of total events, this would not have affected mortality and may have only affected the length of ICU stay.

Adverse event causes resulted from drugs, procedures, surgery, infection and error. Of these, adverse drug events, procedural complications and nosocomial infections were strongly associated with length of ICU stay, even when adjusted for severity of illness, mechanical ventilation status and disease classification. Therefore, it is important to prevent these AEs regardless of admission status or diagnosis. For example, implementation of the pain, agitation and delirium guidelines‐based delirium control programme may reduce delirium, which was the most common adverse drug event (Devlin et al., 2018; Trogrlić et al., 2019). Also, drug‐induced hypotension could be prevented if medical staff understand and standardize goal‐oriented circulation management methods (Rivers et al., 2001). Although prevention of adverse drug events is a difficult task, early detection of adverse drug events is important to prevent them from becoming serious. In order to create a new system for early detection, it would be useful to share knowledge of drugs frequently used in the ICU and to enact team‐wide checks for major adverse reactions when administering drugs. In addition, a multi‐component educational intervention that includes knowledge and practice for nurses could likely improve knowledge and adherence to the Standard Precaution Guidelines, thereby reducing nosocomial infections (Gomarverdi et al., 2019). Other patient safety interventions, such as increased support staff, interdisciplinary team interventions, clinical pathways, and catheter reminder and stop orders, have also been reported to be effective (Zegers et al., 2016). However, single preventative interventions are not enough to reduce AEs with multiple causes and, thus, in the future, a comprehensive AE prevention bundle for adverse drug events, procedural complications, surgical complications and nosocomial infections is critically needed. The implementation of regular surveillance systems for AEs that affect patient outcomes may also improve the perception of patient safety among medical staff since, in Japan, patient safety tends to focus on medical errors while knowledge and practice of AEs and their countermeasures are lacking.

This study has several limitations. First, since the data were collected retrospectively based on medical charts, unrecorded AEs would have been excluded from the analysis and the causal relationship between AE and length of ICU stay thus remains unclear. A prospective study using combinations of conventional methods, such as GTT, direct observation and voluntary reporting (Thomas & Petersen, 2003), although costly in time and funding, might be the most suitable for future studies. Trigger tools may be ideal for this purpose as they are both time and cost‐effective with higher sensitivity than conventional methods (Classen et al., 2011; Naessens et al., 2009). Second, these results may not extrapolate to regional hospitals or other organizations because our research was conducted at a single ICU of one university hospital. It would thus be necessary to increase the number of study facilities in the future. Third, there may have been a selection bias because we did not survey all admitted patients. However, this bias was minimized by random sampling, a method that has been performed since the Harvard Medical Practice Study (Brennan et al., 1991), and was also reported with GTT (Classen et al., 2011; Naessens et al., 2009). Random selection is a recommended sampling approach sufficient to observe the incidence of AEs and temporal changes (Griffin & Resar, 2009). Fourth, since AEs are classified by GTT as physical injuries, psychological suffering (such as anxiety, depression and fear) was not judged as an AE. Since psychological suffering is a common ICU symptom, GTT should be modified to detect these events (Puntillo et al., 2010). Fifth, judgments of preventability depended on the subjectivity of the reviewer as there was no consensus process in the secondary review. However, we tried to maintain objectivity by defining and scaling preventability.

## 5 CONCLUSIONS

Adverse events were common in the Japanese ICU, and most adverse events were classified as “temporary harm.” AEs were associated with length of ICU stay, and in particular, adverse drug events, procedural complications and nosocomial infections were strongly associated with length of ICU stay.

## CONFLICT OF INTEREST

The authors declare that they have no conflicts of interest.

## AUTHOR CONTRIBUTIONS

GA, AO and HS: Study design. GA, AO, HS, CO, CH, MO and TH: Data collection. GA and AO: Statistical analysis. GA: Manuscript documentation. AO and HS: Revision. NS and YI: Conceptualization. All authors read and approved the final manuscript.

## Data Availability

The data sets used and/or analysed during the current study are available from the corresponding author on reasonable request.

## APPENDIX 1

Changes over time in the incidence of adverse events and disease severity

The incidence of adverse events and disease severity are shown as changes per month from April 2016 to March 2017. A, B and C show the incidence of adverse events per 1000 patient‐days, per 100 admissions and the proportion of patients with adverse events respectively. The solid line shows the transition of the mean value for each month. The dotted line shows the mean overall value. D shows the box plot of the Acute Physiology and Chronic Health Evaluation II score as disease severity for each month. AE, adverse event; APACHE, Acute Physiology and Chronic Health Evaluation.

## APPENDIX 2

Severity and preventability of adverse events

Preventable and non‐preventable adverse events categorized between E and I. Category E is temporary harm requiring intervention, category F is temporary harm requiring initial or prolonged hospitalization, category G is permanent harm, category H is life‐threatening harm, and category I is harm causing or contributing to death. AE, adverse event.

## APPENDIX 3

Examples of adverse events with high severity

**Table:**

Cause	Severity	Preventability	Event description
Therapeutic error	Permanent	More than 50%	Tooth damage caused by the use of mouth openers during oral care.
Adverse drug event	Life‐threatening	Less than 50%	A new cerebral haemorrhage occurred due to the use of anticoagulants following the implementation of an extracorporeal circulation device.
Procedural complication	Death	Virtually no evidence	After glycerine enema, the patient developed intestinal perforation and died from septic shock.
Nosocomial infection	Death	Less than 50%	A tracheostomy was performed, but the infection at the tracheostomy site spread and the patient died of mediastinitis.

## APPENDIX 4

Adverse event category

**Table:**

Cause	Type of adverse events	*N* = 284	
		*N*	(%)
Drug (adverse drug event)		119	(42)
	Delirium	56	
	Hypotension	21	
	Nausea or vomiting	14	
	Allergic reaction	8	
	Over sedation	7	
	Haemorrhage	4	
	Coagulation abnormality	4	
	Phlebitis	3	
	Hyperglycaemia	1	
	Asthma	1	
Procedure (procedural complication)		67	(24)
	Skin tear	20	
	Pressure ulcer	12	
	Medical device related pressure ulcer	6	
	Catheter complication	5	
	Hypotension	5	
	Haemorrhage	5	
	Laceration or organ injury	3	
	Pneumothorax	2	
	Blisters	2	
	Cardiac arrest	1	
	Hematoma	1	
	Pulmonary embolus	1	
	Respiratory distress	1	
	Nerve injury	1	
	Vascular injury	1	
	Venous thromboembolism	1	
Surgery (surgical complication)		63	(22)
	Postoperative haemorrhage	33	
	Hypotension	13	
	Respiratory distress	9	
	Arrhythmias	4	
	Shock	1	
	Stroke	1	
	Pneumothorax	1	
	Atelectasis	1	
Nosocomial infection		26	(9)
	Pneumonia, not ventilator related	8	
	Ventilator‐associated pneumonia	6	
	Catheter‐related blood stream infection	5	
	Surgical‐site infection	4	
	Sepsis or bacteraemia unrelated to catheter	2	
	Urinary tract infection	1	
Therapeutic error		5	(2)
	Catheter unplanned removal	2	
	Endotracheal tube unplanned removal	1	
	Laceration or organ injury	1	
	Hypotension	1	
Diagnostic error		4	(1)
	Respiratory distress	2	
	Need for reintubation	2	
